# Fc γ receptor IIIa/CD16a processing correlates with the expression of glycan-related genes in human natural killer cells

**DOI:** 10.1074/jbc.RA120.015516

**Published:** 2020-12-16

**Authors:** Kashyap R. Patel, Maria C. Rodriguez Benavente, W. Walter Lorenz, Emily M. Mace, Adam W. Barb

**Affiliations:** 1 Department of Biochemistry, Biophysics and Molecular Biology, Iowa State University, Ames, Iowa, USA; 2 Department of Biochemistry and Molecular Biology, University of Georgia, Athens, Georgia, USA; 3 Georgia Genomics and Bioinformatics Core and Institute of Bioinformatics, University of Georgia, Athens, Georgia, USA; 4 Department of Pediatrics, Columbia University Irving Medical Center, New York, New York, USA; 5 Complex Carbohydrate Research Center, University of Georgia, Athens, Georgia, USA

**Keywords:** natural killer cells (NK cells), Fc-gamma receptor, N-linked glycosylation, gene expression, glycoproteomics, YTS, NK-92, FcγR, Fc γ receptor, LacNAc, N-acetyllactosamine, NK, natural killer, PNGaseF, peptide N-glycosidase F

## Abstract

Many therapeutic monoclonal antibodies require binding to Fc γ receptors (FcγRs) for full effect and increasing the binding affinity increases efficacy. Preeminent among the five activating human FcγRs is FcγRIIIa/CD16a expressed by natural killer (NK) cells. CD16a is heavily processed, and recent reports indicate that the composition of the five CD16a asparagine(N)-linked carbohydrates (glycans) impacts affinity. These observations indicate that specific manipulation of CD16a N-glycan composition in CD16a-expressing effector cells including NK cells may improve treatment efficacy. However, it is unclear if modifying the expression of select genes that encode processing enzymes in CD16a-expressing effector cells is sufficient to affect N-glycan composition. We identified substantial processing differences using a glycoproteomics approach by comparing CD16a isolated from two NK cell lines, NK92 and YTS, with CD16a expressed by HEK293F cells and previous reports of CD16a from primary NK cells. Gene expression profiling by RNA-Seq and qRT-PCR revealed expression levels for glycan-modifying genes that correlated with CD16a glycan composition. These results identified a high degree of variability between the processing of the same human protein by different human cell types. N-glycan processing correlated with the expression of glycan-modifying genes and thus explained the substantial differences in CD16a processing by NK cells of different origins.

The antibody-binding Fc γ receptors (FcγRs) capture target tissue through a linking antibody to elicit a cytotoxic cell-mediated response. Of the five activating human FcγRs, FcγRIIIa/CD16a expressed by natural killer (NK) cells appears predominant for clearing cancerous cells following the administration of a therapeutic monoclonal antibody (mAb) such as rituximab (1). Accordingly, the therapeutic potential of expressing the antibody-binding Fc γ receptor IIIa (FcγRIIIa/CD16a) on a cultured NK cell line, NK92, alone or fused to the signaling Fc ε receptor γ chain is under active investigation (2, 3). These efforts relate to the recent development of engineered lymphocytes with designed receptors as therapeutic options for late-stage disease, including chimeric antigen receptor T cells and chimeric antigen receptor NK cells (4, 5, 6, 7, 8). The success of these strategies in total provides a validated mechanism to administer engineered transmembrane receptors, opening the possibility of developing engineered FcγRs to enhance many treatments. Recent reports using mutation to prevent activation-induced CD16a shedding or fusing the high affinity FcγRI/CD64 extracellular domain with the CD16a transmembrane and intracellular domains further highlight the potential of engineered FcγRs (9, 10).

CD16a is expressed by most people as the only FcγR on NK cells and is required for NK cell antibody-dependent cell-mediated cytotoxicity (11). CD16a also exhibits substantial structural and functional variability. Two predominant CD16a polymorphic variants provide either greater (V158) or weaker (F158) antibody binding affinity (12, 13, 14). Other mechanisms to increase affinity exist, including altering posttranslation modifications that may be more widely tolerated than engineered polypeptides. The extracellular domain of CD16a contains five asparagine(N)-linked carbohydrates (glycans) at positions N38, N45, N74, N162, and N169 (15, 16) (Fig. 1*A*). Of the five sites, N-glycans at N162 and N45 directly contribute to high-affinity antibody binding and protein stability *in vitro* (17). Manipulating the CD16a N162-glycan composition increases IgG binding affinity, which correlates with mAb efficacy. Hayes and coworkers showed that removing N-acetylneuraminic acid residues from the CD16a N-glycan termini increased binding affinity (12, 18), but later reports showed that the presence of a Man5 oligomannose-type glycan at N162 increased affinity over a complex-type biantennary N-glycan at the same site (17, 19, 20). An analysis of the N162-glycan structures from CD16a isolated from primary human NK cells revealed the presence of both high-affinity and low-affinity CD16a glycoforms (21). Our group also identified multiple other surprising features on CD16a purified from primary NK cells including the presence of hybrid-type N-glycans at N45 and extensive processing including N-acetyllactosamine (LacNAc) repeats at the N38 and N74 sites (Fig. 1*A*).

**Figure 1:**
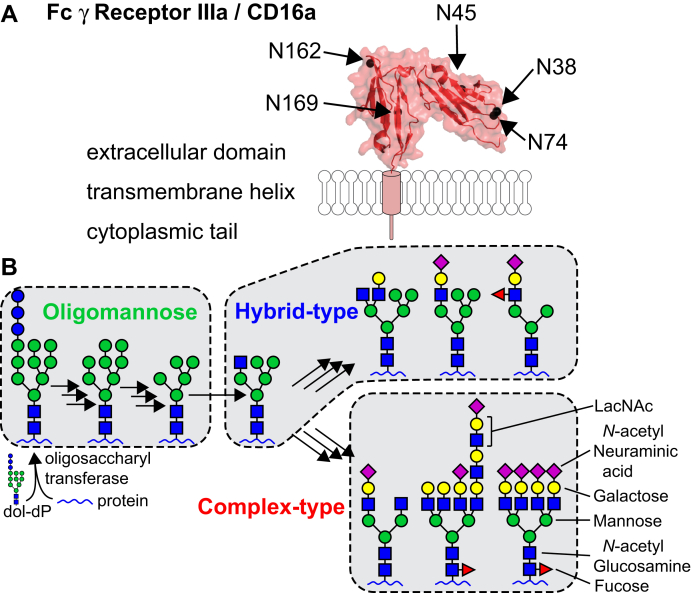
**Human N-glycosylation heterogeneity and the CD16a receptor.***A*, human Fc gamma receptor IIIa/CD16a contains five N-glycosylation sites on the extracellular domain. *B*, N-glycans are attached to the nascent polypeptide chain during import into the lumen of the endoplasmic reticulum. The transferred 14-residue oligosaccharide is trimmed then elongated during protein secretion. The result of this template-independent processing is a potential for considerable N-glycan heterogeneity, although complex-type glycoforms are predominant on serum proteins.

N-Glycans are synthesized without a template, resulting in the formation of oligomannose, hybrid and complex type N-glycan structures that can all occur on the same protein (Fig. 1*B*) (22). Compositional heterogeneity originates from competing processing pathways, incomplete processing by hydrolases and glycosyltransferases, variable enzyme and substrate concentrations, enzyme–substrate exposure time, substrate affinities, accessibility to the glycosylation site, as well as the physiological state of the cell (22). Thus, it is unclear which parameters must be tuned to successfully engineer lymphocyte glycans. An ability to identify and modulate carbohydrate structures likewise provides the ability to prevent the formation of potentially undesirable structures, including oligomannose-type N-glycans on biotherapeutics that are rapidly cleared through binding the mannose receptor (23).

Although many possible factors affect glycan composition, the presence of specific surface carbohydrate structures correlated with the expression of transcripts encoding required glycan-modifying enzymes in mouse stem cells and other systems (24, 25). It is currently unknown if CD16a glycans expressed by human NK cells are as sensitive to gene expression levels. Here we describe the glycogene expression profiles of primary and cultured cell lines including two that exhibit an NK cell phenotype, are widely used to investigate NK cell function, and are candidates for allogenic transplantation (26). These results are compared with the high-resolution mass spectrometry–based glycoproteomics analysis of CD16a expressed by two NK cell lines and HEK293 cells and compared with a previous characterization of CD16a from primary human NK cells (21).

## Results

### Differential mobility of purified CD16a

We analyzed CD16a mobility using SDS-PAGE. Slower mobility associates with greater processing for many mammalian N-glycoproteins. We immunoprecipitated full-length CD16a containing the extracellular, transmembrane, and intracellular domains from suspension HEK293F, NK92, and YTS cells. The NK92 cell line, established from a 50-year-old male patient with non-Hodgkin's lymphoma, was recently engineered to express CD16a (27, 28, 29, 30). The YTS cell line originated from a 15-year-old male with acute lymphoblastic lymphoma and thymoma and was later transformed to express CD16a using the cytomegalovirus promoter (31). Although there are developmental differences between both cell lines, both NK92 and YTS cells perform antibody-dependent cell-mediated cytotoxicity once transformed with CD16a (32).

**Figure 2:**
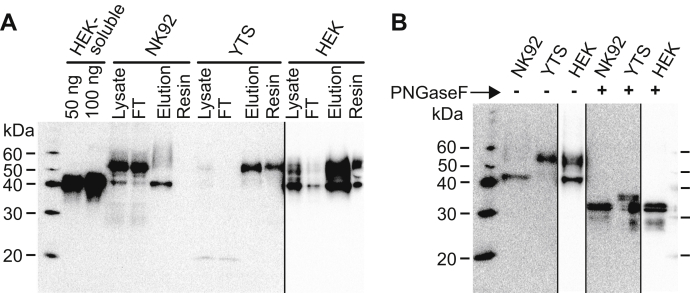
**Human CD16a purified from three difference cell lines.***A*, isolation and purification of CD16a from two human natural killer cell lines (NK92 and YTS) and two protein forms, soluble and full length, expressed by human embryonic kidney 293F cells. These images are Western blots detected with an anti-CD16a primary antibody. *B*, digestion with PNGaseF, an enzyme that removes N-glycans, increases the mobility of each protein. *Black vertical lines* demarcate individual sections of the blot obtained with altered exposure periods to allow comparison of the most intense features. The final images were produced from multiple images of the same blot that differed only by exposure time.

A Western blot, detected with a different anti-CD16a antibody than that used for immunoprecipitation, showed differences in the mobility of CD16a from HEK, YTS, and NK92 cells (Fig. 2*A*). These results are representative of multiple independent experiments. The following purification enriched CD16a. The YTS purification showed a weak band at ∼50 kDa in the lysate that is depleted in the flow-through fraction and enriched in the elution. The mobility of YTS CD16a was less than the mobility of the truncated CD16a protein containing only the extracellular domain (HEK soluble) that lacks 7 kDa corresponding to the transmembrane and intracellular domains.

The full-length CD16a purified from HEK and NK92 cells revealed different features. NK92 cells showed a sharp dark band at ∼50 kDa that did not purify. Because this band was recognized by the detection antibody but not the immunoprecipitation antibody we do not regard this material as properly folded CD16a and did not include it in the study (Fig. 2*A*). HEK and NK92 CD16a showed two distinct purifying bands, one at 40 kDa and another diffuse band >50 kDa (Fig. 2*A*). The lower 40-kDa band was not observed with CD16a from YTS cells. It is possible that the lower band represents unprocessed CD16a. To test this hypothesis, we treated the full-length purified CD16a fragments with peptide N-glycosidase F (PNGaseF) that removes N-glycans by cleaving the linkage to the side-chain asparagine N atom. This treatment collapsed the diffuse 50- to 60-kDa band and the 40-kDa band observed with the HEK and NK92 CD16a revealing a doublet at ∼31 kDa (Fig. 2*B*). Thus, the 40-kDa bands observed in Figure 2*A* do not correspond to non-N-glycosylated CD16a but likely represent CD16a with incompletely processed N-glycans. The presence of complex-type N-glycans further reduces mobility upon comparison with the same protein with the less processed oligomannose N-glycans in support of this possibility (Fig. S1). It is also possible that the lower 40-kDa band observed with CD16a from HEK and NK92 cells lacks one or more, but not all, N-glycans. It is surprising that, PNGaseF-digested CD16a from YTS cells revealed a second doublet at a greater molecular weight (∼34 kDa; Fig. 2*B*). It is possible that YTS CD16a contains an additional non-N-glycan modification or that a protein complex preserved during detergent lysis shielded one or more N-glycans from PNGaseF digestion.

It is notable that each PNGaseF-digested protein migrated as a doublet (Fig. 2*B*); our laboratory observed a similar doublet for PNGaseF-treated CD16a purified from primary human NK cells (33). Electrospray ionization–MS/MS analysis of CD16a did not reveal the presence of an additional modification that could explain this result (data not shown). The apparent mobility differences could be due to partial proteolysis or modification of a portion proximal to the transmembrane domain; we have been unable to identify peptides from this region of the protein in MS-based analyses (21, 33).

In summary, these results demonstrate substantial processing differences between the full-length CD16a expressed by three different mammalian cell lines.

### High-resolution analysis of CD16a glycopeptides

High-resolution glycoproteomics allows for site-specific identification of intact N-glycopeptides, providing a measure of N-glycan processing at each site. Proteolysis of the purified CD16a samples generated glycopeptides that contained a single N-glycan, allowing assignment of individual glycoforms to a single site within the protein. We identified a total of 276 unique N-glycopeptides that displayed 121 individual N-glycan species from the four different CD16a samples (Table 1). The numbers of individual glycopeptides from each source ranged from 60 for N162 of the HEK CD16a to zero for the N169 site of YTS CD16a. The average absolute mass error for the glycopeptides was <0.005 Da (Table 1).

**Table 1:** Distinct glycopeptides identified for each sample

N-glycosylation site	HEK-soluble	HEK	NK92	YTS	Total	Abs mass error ± SD (Da)
N38	38	44	7	12	68	0.0056 ± 0.0087
N45	34	44	18	18	50	0.0040 ± 0.0039
N74	30	29	6	6	52	0.0046 ± 0.0048
N162	53	60	15	10	73	0.0049 ± 0.0037
N169	28	13	8	0	33	0.0042 ± 0.0032
Mass error (all data)						0.0047 ± 0.0054
# of Distinct N-glycans	86	99	30	36	121	

**Figure 3:**
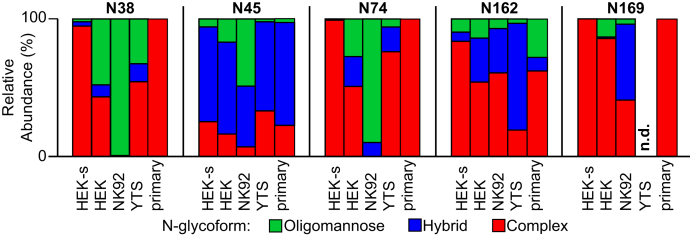
**Relative abundance of N-glycan types at each CD16a N-glycosylation site.** “Primary” indicates average compositions from CD16a isolated from the primary NK cells of five donors (21). n.d., not observed.

We observed striking differences in the processing at individual CD16a N-glycan sites resulting from expression with different cell types (Fig. 3). For example, N38 from HEK-soluble CD16a contained nearly exclusively complex-type N-glycoforms in contrast to NK92, which showed 99.4% oligomannose types. HEK and YTS CD16a reveal comparable N38 profiles with ∼40% oligomannose, ∼10% hybrid, and ∼50% complex types. Comparable processing across all cell lines likewise emerged for the N74 site, which is proximal to N38 in the folded protein (Fig. 1). It is surprising that the processing at N45 on all cell types was comparable with that observed for primary human NK cells with CD16a containing 60% to 70% hybrid forms, except CD16a from NK92 cells, which showed hybrid but a larger percentage of oligomannose forms. The N162 site exhibited substantial variability with HEK-soluble displaying 83% complex types and YTS with 78% hybrid types. Although we observed fewer N169-containing glycopeptides, likely owing to incomplete proteolysis that is required to separate this peptide from the transmembrane domain for the full-length proteins, HEK and HEK-soluble displayed primarily complex types and NK92 cells showed predominantly hybrid types.

**Figure 4:**
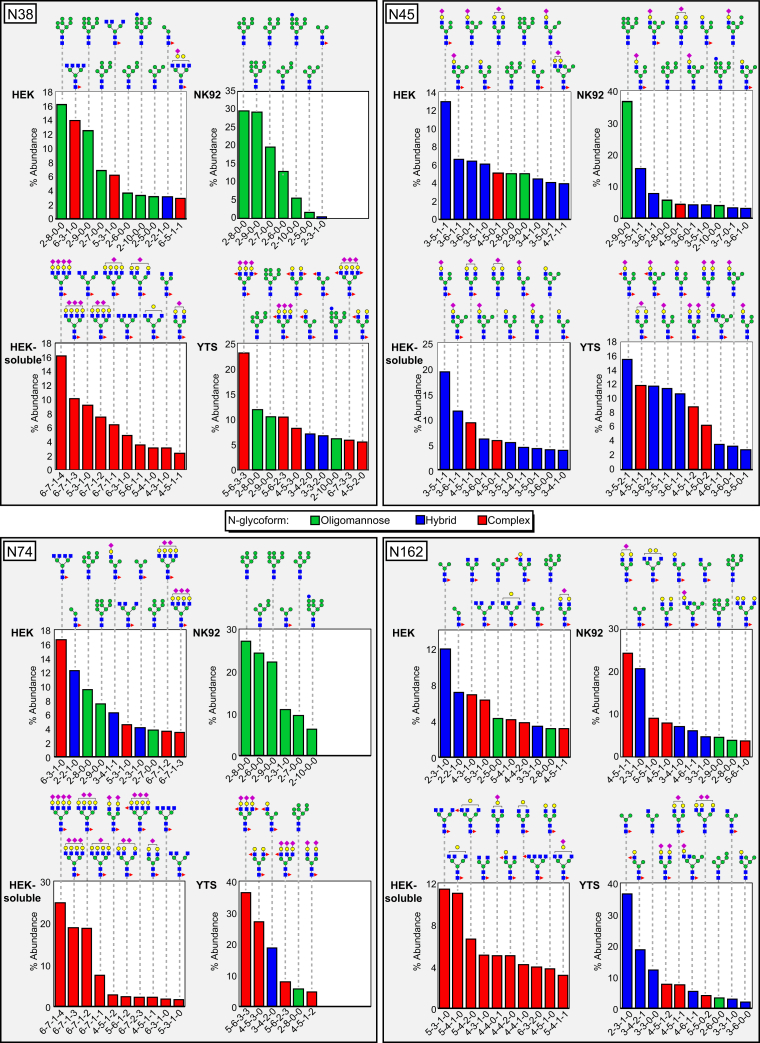
**The 10 most abundant CD16a glycoforms at the N38, N45, N74, and N162 sites.** The cartoon diagrams represent one possible configuration; isobaric ions were not distinguished. The composition of each N-glycan is provided below the chart with numbers of N-acetylhexosamine, hexose, deoxyhexose, and N-acetylneuraminic acid residues, respectively.

An examination of the most abundant glycoforms at each site likewise revealed substantial differences between the CD16a expressed by each cell line (Figs. 4 and S2). N38 and N74 from HEK-soluble CD16a appear as the most processed sites with a high degree of branching and branch processing with the most abundant forms showing complete sialylation. However, none of these expression hosts generated N38 and N74 glycans with LacNAc repeats that are a predominant feature of CD16a expressed in primary monocytes and NK cells (21, 33). These two sites from HEK and NK92 CD16a show a high percentage of oligomannose and partially processed forms, with exclusively oligomannose forms from NK92 cells.

It is notable that YTS CD16a, but not NK92 CD16a, shows a high degree of terminal branch processing that is revealed by the extensive galactosylation, sialylation, and branch fucosylation (Fig. 4). CD16a isolated from primary NK cells likewise showed a high degree of galactosylation and sialylation at the branch termini with more modest fucosylation (19, 21). CD16a isolated from NK92 cells showed the least N-glycan processing, especially at the N38 and N74 sites, lacking many of the features observed with CD16a from primary NK cells (19, 21). In addition to the aforementioned high levels of oligomannose glycoforms from NK92, the few complex-type N-glycans at N45 and N162 contained substantially less branching than CD16a from the four other sources.

The glycoproteomics strongly supported the SDS-PAGE mobility analysis and demonstrated the appearance of substantial processing differences between CD16a isolated from different human cell lines.

### Glycogene expression profiling

Transcriptional regulation of the enzymes responsible for N-glycan processing is one mechanism to modulate cellular glycosylation. We evaluated the expression of genes that encode glycan-modifying enzymes using RNA-Seq and qRT-PCR with RNA isolated from primary NK cells from three healthy donors in addition to samples taken from the HEK293F, NK92, and YTS cells lines. A principal component analysis of the RNA-Seq data showed the formation of distinct clusters formed by each cell line (Fig. S3). Furthermore, changes in the expression of key transcripts encoding N-glycan-modifying enzymes from each cell line relative to primary NK cells showed a strong correlation with data from the qRT-PCR including an *R*^2^ value of 0.98 and 0.84 comparing changes between methods for HEK293F cells and NK92 cells, respectively (Tables S1 and S2). The correlation of the YTS data was lower, with an *R*^2^ of 0.31; however, this correlation is strongly influenced by a single observation that is expressed at low levels in only two of the seven YTS cell samples and showed no significant difference in the qRT-PCR data (ST8SIA2). Removing this data point increases the correlation to 0.68. We will discuss changes from the RNA-Seq data in the following discussion owing to the high similarity of the RNA-Seq and qRT-PCR and the greater depth of gene coverage in the RNA-Seq data.

A pairwise comparison of the normalized mean count for each transcript revealed thousands of differences between different cell lines. One striking difference was the ∼1000-fold higher expression of the B3GAT1 transcript in primary NK cells as compared with NK92 and YTS cells (Table S1). This enzyme generates the CD57/HNK1 epitope that is a marker of mature NK cells (34). Another striking example was the 450-fold reduction of B3GNT7 expression as well as reduced levels of B3GNT8 and B3GNT2 in all cultured cells that correlated with the complete absence of LacNAc repeats on the N38 and N74 glycans that are prevalent features of CD16a from primary NK cells (Tables 2 and S1) (21). Enzymes encoded by these genes in concert with the B4GALTs synthesize LacNAc structures (35).

**Table 2:** RNA-Seq data for select glycan processing genes from three cell lines reporting the change in expression relative to primary human NK cells

Gene	HEK293F	NK92	YTS	Enzyme product
B3GNT7	− − −	− −	− − −	β1-3GlcNAc in LacNAc repeats
B3GNT8	n.s.	n.s.	− −	β1-3GlcNAc in LacNAc repeats
B4GALT2	++	++	++	Branch β1-4Gal
B4GALT1	− −	−	−	Branch β1-4Gal
FUT9	+++	++	n.s.	Branch (α1-3/4)Fuc
FUT4	n.s.	− −	− − −	Branch (α1-3/4)Fuc
FUT5	n.s.	− −	n.s.	Branch (α1-3/4)Fuc
FUT7	− −	n.s.	++	Branch (α1-3/4)Fuc
MAN2A2	−	− − −	n.s.	GlcNAc Man3 glycoform
MGAT4A	−	− − −	− −	Branch (β1-4GlcNAc to α1-3Man)
MGAT5	− −	n.s.	− − −	Branch (β1-6GlcNAc to α1-6Man)
MGAT3	++	n.s.	n.s.	Bisecting GlcNAc
MGAT5B	++	n.s.	++	Branch (β1-6GlcNAc to α1-6Man)
MGAT1	−	n.s.	n.s.	Branch (GlcNAc Man5)
ST3GAL6	++	++	− −	α2,3 Neu5Ac
ST6GAL1	− −	− −	−	α2,6 Neu5Ac

“−” indicates a decrease, “+” an increase, and “n.s.” is not significant at *p*-adj ≤ 0.05.

Complete data with fold change and significance values are shown in Table S1.

NK92 cells showed a >100-fold reduction in MAN2A2 expression, a gene that encodes a mannosidase required to generate complex-type N-glycans. Oligomannose glycans are absent from the CD16a N38, N74, and N169 sites at the surface of primary human NK cells, indicating the higher activity of this enzyme (21). The decreased MAN2A2 transcript abundance from NK92 cells matched the increased abundance of oligomannose-type N38, N45, and N74-glycans on CD16a. The HEK293F, NK92, and YTS cells generally expressed lower levels of key mannosidases, likely contributing to the accumulation of oligomannose N-glycoforms (Fig. 3).

YTS cells showed decreases of 125- and 31-fold for MGAT5 and MGAT4A transcripts encoding branching enzymes, respectively, that correlated with a reduction in branched, complex-type glycoforms at N38 and N74 of the YTS CD16a. Highly branched glycans are prevalent features of the CD16a N38 and N74 glycans isolated from primary NK cells, likely the result of MGAT4A and MGAT5 activity (21). The N-glycan composition analysis suggests that the 17-fold increase of MGAT5B that encodes a related branching enzyme was insufficient to compensate for decreased MGAT5 and MGAT4A expression. NK92 cells similarly expressed MGAT4A at >500-fold reduction relative to primary cells, whereas HEK cells showed a 6-fold reduction in MGAT4A. Of interest, it is believed that the enzymatic product of the MGAT5 transcript is required for LacNAc addition by the B3GNTs described above, further reducing the potential for LacNAc incorporation in CD16a expressed by HEK293F and YTS cells.

YTS cells showed a 20-fold increase of FUT7 transcripts that matched the observation of outer glycan branch fucosylation on the N38, N45, and N74 glycans compared with primary NK cells that showed less branch fucosylation (21, 33). FUT7 is the only branch fucosyltransferase-encoding gene that increases compared with primary NK cells (Tables 2 and S1). A 20-fold decrease in FUT7 expression by HEK293 cells may explain the near absence of branch fucosylation in CD16a purified from HEK293 cells. It is surprising that the glycan composition was not compensated by a 332-fold increase in FUT9 expression. It is possible that the abundance of sialylated branch termini inhibited FUT9 activity as described previously, whereas FUT7 selectively modifies sialylated branches (36).

Expression of the ST3GAL6 sialyltransferase gene increased by 62- and 31-fold in HEK293F and NK92 cells, respectively, but this was insufficient to fully sialylate many galactose-terminated branches, unlike CD16a from primary cells that was highly sialylated with few unmodified galactose residues. HEK293F and NK92 cells also showed substantial reductions in other sialyltransferase transcript levels, including a respective 10- and 15-fold reduction of ST6GAL1 expression. YTS cells showed an increase in ST3GAL1, 4, and 3 expression but reductions in ST6GAL1 and ST3GAL5.

Gene expression profiling demonstrates substantial differences in the expression of glycan-modifying enzymes present in these four cell types that are consistent with compositional changes observed from the glycoproteomics analysis.

## Discussion

Here we identified substantial differences distinguishing NK cells of different origins. It is notable that CD16a from NK92 cells contained a high percentage of minimally remodeled N-glycans that may result from improper protein processing during secretion. This result demonstrates that NK92 cells do not sufficiently process CD16a and thus do not display CD16a with glycoforms on the surface that are similar to primary NK cells. This incomplete processing may affect antibody binding affinity or other yet to be described CD16a functions at the cell surface, if CD16a behavior on the cell surface is accurately recapitulated by CD16a affinity measurements that indicated the strong connection between CD16a processing and high affinity (17, 18, 19). Furthermore, the high levels of oligomannose N-glycans, particularly at N38 and N74, are known as potent ligands for the macrophage mannose receptor and DC-SIGN and could limit infused NK92 cell availability or cause other complications (37). The origin of the processing deficits is likely related to the reduced mannosidase expression in these cells including the 100-fold reduction in MAN2A2 activity (Table 2). YTS cells likewise showed CD16a processing deviations compared with primary NK cells including an increased branch fucosylation and an absence of poly-LacNAc structures. Lastly, HEK293F-expressed CD16a showed substantial differences from that of primary NK cells. The HEK293F-expressed material failed to accurately recapitulate CD16a processing from primary NK cells and these differences should be considered when performing *in vitro* CD16a binding assays to evaluate mAbs.

The glycan processing differences were closely mirrored by changes in the expression levels for glycan-modifying enzymes. A similar connection was observed for mucosal cells and murine embryonic stem cells (24, 25), indicating that targeting the expression of individual glycan-modifying enzymes is expected to impact CD16a composition at the cell surface. This leads to the strong possibility that modifications to limit CD16a N162 glycan processing will produce tight binding glycoforms on the cell surface, given the aforementioned role of CD16a glycan composition on modulating antibody-binding affinity.

**Figure 5:**
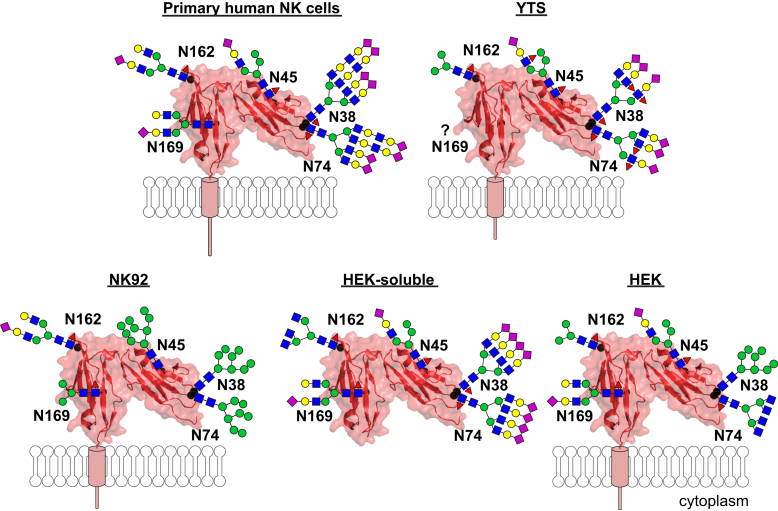
**Models of the most abundant CD16a N-glycoforms.** The cartoons are roughly scaled to the appropriate size. Primary human NK cell N-glycans were determined previously (21).

Although neither the YTS nor NK92 NK cells processed CD16a in a manner identical to primary NK cells, the YTS cells showed the highest degree of similarity among the full-length CD16a proteins from the three different cell types (Fig. 5). It is notable that the migration of the processed YTS CD16a was only slightly increased compared with that reported previously for CD16a purified from primary human NK cells that migrated between 53 and 59 kDa (21, 33). The individual N-glycan similarities included a high percentage of hybrid-type N-glycans at N45 and complex-type N-glycans at N38 and N74. The YTS CD16a N162 composition falls within the range of forms associated with primary NK cell CD16a, although such a comparison is limited by the high degree of variability between primary cell donors (21).

These results identify a high degree of variability between the processing of a single human protein by different human cell types. Each cell creates a unique Golgi environment that impacts the processing of the secreted protein, with surprising differences between different cell types despite comparable developmental lineages, although it is not yet known how glycosylation differences impact cell activation. Here we also identify multiple genes potentially suitable for manipulation of expression levels in cultured effector cells, providing Golgi conditions as similar as possible to NK cells in the human body. Furthermore, glycoprotein processing is predominantly mediated by factors specific to each individual cell line, and not based solely on factors intrinsic to the glycoprotein sequence. It is then obvious that cultured cells must exhibit appropriate processing to delineate the functional role of individual modifications in cell function.

One limitation of the present study is detergent lysis that mixes fully and partially processed forms. Although we saw little evidence for underprocessing with primary human NK cells when lysed with detergent (21), imprecise control of transcription and translation resulting from ectopic expression may overwhelm folding and processing during protein secretion and contribute to the high levels of underprocessed forms observed with NK92 and HEK293F CD16a. This result was surprising because NK92 cells are phenotypically NK cells but produced CD16a that was less similar to primary human NK cell CD16a than CD16a produced by HEK293F cells, likely derived from adrenal cells (38). NK92 cells display surface markers and a genetic profile that is more consistent with premature NK cells (39, 40) and also do not provide identical expression levels of Golgi-localized processing enzymes that are comparable with differentiated peripheral NK cells.

## Experimental procedures

All materials were purchased from Millipore-Sigma unless noted otherwise.

### CD3ζ expression vector

The open reading frame encoding CD3ζ was cloned from cDNA obtained from primary human NK cell RNA isolated previously (19). A high-capacity RNA to cDNA kit (Applied Biosystems) was used to reverse transcribe 500 ng of RNA and amplified with AccuPrime Pfx DNA polymerase (Life Technologies, Inc) to generate full-length cDNA. DNA was then amplified to introduce cloning sites (primers F: GAATTCAAAGGACAAGATGAAGTGGAAGGC; R: GGATCCTCATTAGCGAGGGGGCAGGGC). An internal BamHI site was removed with fusion PCR using the previous primers along with two internal primers (Fa: CCTGCTGGACCCCAAACTCT;Ra: AGAGTTTGGGGTCCAGCAGG). DNA encoding CD3ζ with the native signal sequence was cloned into the EcoRI and BamHI sites of the pGEef1Puro vector (a gift from Dr Kelley Moremen, University of Georgia).

### Cell culture and CD16a expression

YTS-CD16 cells were maintained in RPMI medium supplemented with 10% fetal calf serum and 50 U/ml of penicillin/streptomycin, 10 mM Hepes, 1 mM sodium pyruvate, 2 mM L-glutamine, and 1 mM nonessential amino acids (all supplements from Gibco). NK92-CD16 cell lines (American Type Culture Collection) were maintained in Myelocult media (Stemcell Technologies) supplemented with 200 U/ml of IL-2 (Roche) and 50 U/ml of penicillin/streptomycin (Gibco). Cell lines were a gift from Dr Jordan Orange (Columbia University) and were routinely confirmed to be mycoplasma negative. Cell line identity, including CD16 expression, was confirmed by flow cytometry (data not shown). Human embryonic kidney 293F (HEK293F) cells were maintained as described (41). Full-length CD16a (HEK) was coexpressed from the pGEN2-(full length)CD16a vector with a 2× mass excess of the pGEef1Puro-CD3ζ vector using HEK293F cells as described (19). Cell surface expression of CD16a in YTS, NK92, and HEK293F was confirmed by flow cytometry. The soluble extracellular domain of CD16a (HEK-soluble) was expressed in HEK293F cells as described (16).

### CD16a purification and proteolysis

HEK-soluble CD16a was purified from the expression medium using Ni-NTA resin as described (16). Full-length CD16a from NK92, YTS, and HEK cells was extracted with dodecyl maltoside and immunoprecipitated using immobilized 3G8 antibody (19). The denatured protein was proteolyzed with chymotrypsin and Glu-C, alkylated, and enriched for glycopeptides with the ProteoExtract Glycopeptide Enrichment Kit (Millipore Sigma) as described (21). PNGaseF treatment and immunoblotting were conducted as described (33). For immunoprecipitation, cells were lysed and the supernatant was incubated for 1 h at 4 °C with Protein G-Sepharose. The recovered supernatant was later incubated with previously expressed and immobilized 3G8-coupled agarose beads overnight at 4 °C, washed, and CD16 was eluted in 45:55:0.1 (water:acetonitrile:TFA) by centrifugation through a spin column to retain resin. For Western blotting, the eluted samples were resolved on an SDS-PAGE gel and transferred on a polyvinylidene difluoride membrane using the one-step electroblotting system (Thermo Fisher Scientific). The membrane was blocked with 5% dry milk in tris-buffered saline Tween 20 buffer for 1 h at room temperature and incubated with anti-hCD16 (R&D Systems #AF1597, 1:10,000) overnight at 4 °C. The blot was washed multiple times and incubated with secondary anti-goat IgG Alexa Fluor 680 (Invitrogen #A32860, 1:10,000) for 1 h at 4 °C. Blots were imaged after multiple washes with Odyssey Fc Imaging system (LI-COR Biosciences).

### Liquid chromatography/mass spectrometry

Separation and mass analysis were performed as described (13, 19). Data collection parameters are reported according to MIRAGE guidelines (see Supporting Information). Briefly, samples were analyzed using a 75 μm × 20 cm fused silica capillary column/emitter (Agilent Technologies) packed with 3 μm Agilent Zorbax reversed-phase C18 medium using a pressure cell (Next Advance). The column was attached to an EASY nLC-1200 LC system with a Nanospray FlexIon source (Thermo), and the ion transfer tube was heated at 275 °C during operation. All reagents were HPLC grade. The mobile phase was composed of 0.1% formic acid in water (buffer A) and 80% acetonitrile, 0.1% formic acid in water (buffer B). Samples were eluted from 0 to 60 min with a 0% to 35% gradient of buffer B at a flow rate of 300 nl/min. The column was washed with 70% buffer B from 60 to 70 min and with 100% buffer B from 70 to 80 min. The column was equilibrated by passing 20 μl buffer A at 400 bar. A blank run (5 μl buffer A was injected instead of sample) followed each sample run. An electrospray voltage of 2650 V was used for ionization. The separated glycopeptides and peptides were fed by LC into a Q Exactive Hybrid Quadrupole-Orbitrap Mass Spectrometer with a high-energy collisional dissociation fragmentation cell (Thermo). S-lens radiofrequency levels were set at 50%. Glycopeptides were fragmented in using stepped normalized collision energy of 17, 27, and 37 eV. Data were acquired using Xcalibur Software (Version 2.8.1.2806; Thermo) in the positive ion mode. Glycopeptide ions within a scan range of 600 to 2500 m/z and peptide ions within scan range of 266 to 2500 m/z were collected for analysis. The 20 most intense ions in the 80-ms elution window were collected for high-energy collisional dissociation fragmentation. Ion m/z values and intensity were exported and analyzed using the Xcalibur Qual Browser Software (Version 4.0.27.19; Thermo). A glycopeptide peak list was generated from RAW file by exporting m/z values and intensity from a retention time range unique for each glycopeptide. The m/z and intensity values for these glycopeptides are noted in the Supplemental Excel sheet. Raw mass spectra were initially parsed using Byonic (analyses were performed according to (13)). Briefly, the identity of each glycopeptide was verified by matching the observed parent ion mass to theoretical mass and identifying the ion peak corresponding to the peptide with one GlcNAc in MS2 spectra (if available). Glycopeptides were manually validated by comparing retention time to that of known peaks corresponding to the same peptide and analyzing MS2 spectra for characteristic features including glycan oxonium ions, peptide ions, peptide y and b ions, peptide + GlcNAc, and peptide + glycan ions. A custom R script extracted the intensity of the first seven isotopolog peaks of the glycopeptides, and these intensities were summed to calculate the relative abundance of individual glycopeptides. The error range for peak selection was set at 0.015 m/z. The intensities of identical N-glycan species from different peptides containing the same N-glycosylation site were summed for analysis. MS data were deposited to the MassIVE database (massive.ucsd.edu) as accession MSV000085785.

### RNA isolation

Six samples from each cultured cell line were isolated at the University of Georgia (UGA). RNA from 2 × 10^6^ YTS and NK92 cells was extracted using the RNeasy Mini Kit (Qiagen) according to the manufacturer's guidelines. RNA from 5 × 10^5^ HEK293F cells was extracted using the RNeasy Micro Kit (Qiagen). Six total RNA samples were isolated at Iowa State University as described (one for each cultures cell line and three from NK cell donors) (19). The primary NK cell donors were men aged 40, 24, and 35 years, and the resulting samples designated NK00, NK14, and NK15, respectively. The RNA concentration was measured using the Nanodrop2000 spectrophotometer (Thermo Fisher) and the integrity was measured using the Agilent BioAnalyzer, a service provided with the support of the Georgia Genomics and Bioinformatics Core at UGA. Only RNA that had an RNA integrity number higher than 7.0 was considered for further experiments.

### RNA-Seq

The total RNA (RNA integrity number ≥7.0) was submitted to both the Georgia Genomics and Bioinformatics Core at UGA and the DNA Facility at Iowa State University for sequencing. RNA-Seq libraries were synthesized using a QuantSeq 3ʹ mRNA-Seq library kit (Lexogen). Single-read sequencing was performed on Illumina (Illumina) NextSeq 500 (SR75) and HighSeq 3000 (SR100) instruments at UGA and Iowa State University, respectively.

Bioinformatics analyses were performed by UGA's Georgia Genomics and Bioinformatics Core, and computational work was done using the high-performance computational resources at UGA's Georgia Advanced Computing Resource Center. Read quality metrics of raw and processed read data were assessed at each step using FastQC (42). Trimmomatic, version 0.36 (43), was employed to remove residual adapter/index sequences and for quality trimming of reads. Reads with a trimmed length below a 30-base threshold were discarded. Contaminating ribosomal RNA sequences were removed after trimming by alignment with Bowtie2, ver. 2.3.4.1 (44) to a human rRNA reference dataset. Unmapped reads were output and used for subsequent analysis. The *H. sapiens* genome accession, GRCh38/hg38, Dec. 2013, was obtained from the UCSC Genome Browser (45). Trimmed, filtered reads were then mapped against hg38 using the STAR aligner, ver. 2.7.1a (46); HTSeq, ver. 0.9.1 (47) was used to extract raw count data from each BAM file and output files containing gene-to-count information for each sample were concatenated to generate a raw count matrix that was then manually filtered to remove rows containing zero counts or rows whose summation of counts was less than 50. The low-count filtered matrix was used as input for subsequent RNA-Seq expression analyses.

The Bioconductor package DESeq2 (48) was run in the R, ver 3.6.3, computing environment to normalize count data, estimate dispersion, and test for differential expression using negative binomial generalized linear models for analysis of differentially expressed genes. A metadata table was generated containing relevant sample and replicate associations for each experimental grouping. Genes meeting a false discovery rate threshold value ≤0.05 were considered statistically significant. Regularized logarithm (rlog) transformation of the raw count matrix was used for principal component analysis. In addition, as a comparison, the Bioconductor package edgeR (49) was also used to call differentially expressed genes following trimmed mean of M-values normalization of the filtered matrix and comparison of groups via exactTest to calculate genewise expression differences and probability statistics. Raw sequence data are available at NCBI BioProject as entry GSE153738.

### qRT-PCR

First-strand cDNA was synthesized from 0.5 μg total RNA using the RT2 First Strand Kit (Qiagen) according to the manufacturer's instructions. The analysis of 84 genes involved in glycosylation was carried out using a human RT^2^ Profiler PCR Array (cat. # PAHS-046Z; Qiagen) according to the manufacturer's instructions. The array was repeated four times (n = 4) for each cell line with independently isolated RNA. All samples passed QC in the array internal controls and were normalized by arithmetic mean using all five housekeeping genes in the panel (ACTB, B2M, GAPDH, RPLP0, HPRT1). Fold change was calculated by the 2^−ΔΔCT^ method.

## Data availability

MS data are available in the MassIVE database (massive.ucsd.edu) as accession MSV000085785 and the RNA-Seq data are available at NCBI BioProject as entry GSE153738.

## Conflict of interest

The authors declare that they have no conflicts of interest with the contents of this article.
