# Supplementary material for: Fc γ receptor IIIa/CD16a processing correlates with the expression of glycan-related genes in human natural killer cells
Source: J Biol Chem. 2020 Dec 16;296:100183. doi: 10.1074/jbc.RA120.015516 (PMC7948478; doi:10.1074/jbc.RA120.015516)
Supplement: Supplemental file 3 [file mmc3.pdf]

Supporting Information for

Fc γ receptor IIIa / CD16a processing correlates with the expression of glycan-related genes in human natural killer cells

Kashyap R. Patel[1†], Maria C. Rodriguez Benavente[2†], W. Walter Lorenz[3], Emily M. Mace[4], Adam W. Barb[2,5]*

Table S1. RNA-Seq data for N-glycan processing genes from three cell lines reporting the fold change in expression relative to primary human NK cells. "n.s." denotes not significant at p-adj ≤ 0.05.

Gene	HEK293F	p-adj (FDR)	NK92	p-adj (FDR)	YTS	p-adj (FDR)	Magnitude of the maximum change	Enzyme Product
B3GAT1	0.071	1.8E-04	0.0010	5.1E-16	9.3E-4	3.6E-16	1081	HNK1 synthesis
B3GNT7	0.0024	2.0E-22	0.083	1E-04	0.0022	2.1E-22	458.7	LacNAc repeats
B3GNT8	n.s.	n.s.	n.s.	n.s.	0.024	2.0E-02	40.9	LacNAc repeats
B3GNT2	5.3	2.5E-18	0.61	2E-02	n.s.	n.s.	5.3	LacNAc repeats
B4GALNT3	29	2.0E-07	80	9E-12	n.s.	n.s.	79.7	Branch GalNAc
B4GALNT4	77	1.0E-08	n.s.	n.s.	n.s.	n.s.	76.9	Branch GalNAc
B4GALT2	19	2.2E-22	19	1E-21	14	1.2E-17	19.3	Branch Gal
B4GALT1	0.067	7.9E-18	0.11	6E-13	0.12	7.1E-12	14.9	Branch Gal
B4GALT4	n.s.	n.s.	0.19	3E-02	0.17	1.8E-02	6.1	Branch Gal
B4GALT3	0.72	4.9E-02	1.4	5E-02	n.s.	n.s.	1.4	Branch Gal
FUT9	332	5.9E-05	24	5E-02	n.s.	n.s.	332.0	Branch Fuc (α1-3/4)
FUT4	n.s.	n.s.	0.068	2E-03	0.0077	3.8E-07	129.9	Branch Fuc (α1-3/4)
FUT5	n.s.	n.s.	0.044	4E-02	n.s.	n.s.	22.6	Branch Fuc (α1-3/4)
FUT7	0.048	1.2E-04	n.s.	n.s.	20	1.2E-05	20.7	Branch Fuc (α1-3/4)
FUT11	0.12	5.4E-10	n.s.	n.s.	n.s.	n.s.	8.3	Branch Fuc (α1-3/4)
FUT1	6.6	2.9E-03	n.s.	n.s.	n.s.	n.s.	6.6	Branch Fuc (α1-2)
FUT8	n.s.	n.s.	n.s.	n.s.	3.2	2.1E-05	3.2	Core Fuc (α1-6)
MAN2A2	0.38	4.4E-02	0.009	2E-25	n.s.	n.s.	111.8	GlcNAc Man3 glycoform
MAN1A2	0.26	5.5E-10	0.32	2E-07	0.43	2.7E-04	3.9	Man5 glycoform
MAN1A1	n.s.	n.s.	n.s.	n.s.	0.35	1.1E-03	2.9	Man5 glycoform
MGAT4A	0.16	1.4E-04	0.0018	7E-37	0.032	2.7E-13	561.4	Branch (β1-4GlcNAc to α1-3Man)
MGAT5	n.s.	n.s.	n.s.	n.s.	0.008	3.7E-35	124.6	Branch (β1-6GlcNAc to α1-6Man)
MGAT3	19	1.5E-03	n.s.	n.s.	n.s.	n.s.	19.1	bisecting GlcNAc
MGAT5B	16	3.8E-03	n.s.	n.s.	17	3.5E-03	16.7	Branch (β1-6GlcNAc to α1-6Man)
MGAT1	0.23	2.9E-09	n.s.	n.s.	n.s.	n.s.	4.4	Branch (GlcNAc Man5)
ST3GAL6	62	1.1E-06	31	1E-04	0.065	1.4E-02	62.4	α2,3 Neu5Ac
ST6GAL1	0.095	7.3E-11	0.066	7E-14	0.28	1.2E-03	15.2	α2,6 Neu5Ac
ST3GAL5	0.13	3.1E-12	0.44	1E-02	0.25	5.2E-06	7.7	α2,3 Neu5Ac
ST3GAL4	0.32	3.1E-04	0.19	2E-07	4.8	4.1E-07	5.2	α2,3 Neu5Ac
ST3GAL1	0.22	1.7E-11	n.s.	n.s.	1.8	1.8E-02	4.6	α2,3 Neu5Ac
ST3GAL3	n.s.	n.s.	n.s.	n.s.	2.4	4.7E-02	2.4	α2,3 Neu5Ac
ST3GAL2	0.45	1.9E-06	0.61	6E-03	n.s.	n.s.	2.2	α2,3 Neu5Ac
ST6GALNAC6	0.073	2.1E-03	0.027	4E-05	0.025	3.5E-05	40.4	α2,6 Neu5Ac to GalNAc
ST6GALNAC3	23	4.1E-06	n.s.	n.s.	31	5.7E-07	30.6	α2,6 Neu5Ac to GalNAc
ST6GALNAC2	8.8	2.0E-03	n.s.	n.s.	0.12	8.1E-03	8.8	α2,6 Neu5Ac to GalNAc
ST8SIA6	0.043	2.8E-10	0.0018	1E-25	0.13	7.1E-05	563.2	polysialic acid
ST8SIA2	377	5.9E-06	n.s.	n.s.	23	3.1E-02	376.9	polysialic acid
ST8SIA5	49	7.1E-03	n.s.	n.s.	n.s.	n.s.	49.0	polysialic acid
ST8SIA4	0.1	7.6E-07	n.s.	n.s.	n.s.	n.s.	7.1	polysialic acid

Table S2. qPCR data for N-glycan processing genes from three cell lines reporting the fold change in expression relative to primary human NK cells. "n.s." denotes not significant at $p \leq 0.05$.

Gene	HEK293F	p-value	NK92	p-value	YTS	p-value	Magnitude of the maximum change	Enzyme Product
AGA	0.36	8.8E-03	0.12	1.6E-03	0.41	1.5E-02	8.3	catabolism of N-linked oligosaccharides
B3GNT8	0.53	1.4E-02	0.11	2.8E-04	0.05	2.1E-04	20.0	β1-3 GlcNAc in LacNAc repeats
B3GNT3	n.s.	n.s.	0.24	2.2E-05	n.s.	n.s.	4.3	β1-3 GlcNAc in LacNAc repeats
B3GNT2	0.29	1.2E-03	0.57	2.7E-04	0.4	4.0E-04	3.4	β1-3 GlcNAc in LacNAc repeats
B4GALT2	37.09	1.4E-04	5.33	5.0E-06	3.34	1.4E-02	37.1	Branch β1-4Gal
B4GALT1	0.12	5.0E-06	0.06	2.0E-06	0.06	2.0E-06	16.7	Branch β1-4Gal
B4GALT3	n.s.	n.s.	0.52	2.4E-05	0.25	2.1E-05	4.0	Branch β1-4Gal
EDEM1	0.29	2.0E-06	0.62	1.8E-03	0.44	1.0E-06	3.4	ER-associated N-glycoprotein degradation
EDEM2	0.53	1.2E-03	0.41	1.7E-05	0.55	3.6E-03	2.4	ER-associated N-glycoprotein degradation
EDEM3	n.s.	n.s.	n.s.	n.s.	n.s.	n.s.	n.s.	ER-associated N-glycoprotein degradation
FUCA2	6.84	5.7E-04	0.35	3.1E-03	0.01	3.5E-04	100.0	α1-6Fuc hydrolysis from reducing-end GlcNAc
FUCA1	3.06	2.3E-03	0.38	6.9E-04	0.27	6.0E-06	3.7	α1-6Fuc hydrolysis from reducing-end GlcNAc
FUT11	0.17	1.0E-06	0.14	1.0E-06	0.16	1.0E-06	7.1	Branch (α1-3/4)Fuc
FUT8	n.s.	n.s.	0.24	9.0E-06	0.64	2.2E-02	4.2	Core (α1-6)Fuc
GANAB	1.86	3.7E-02	0.82	3.3E-02	0.68	1.4E-03	1.9	ER-associated Glu hydrolysis
GLB1	4.34	2.7E-04	0.53	1.9E-03	0.33	5.9E-04	4.3	terminal bGal hydrolysis
GNPTAB	0.12	6.3E-03	0.19	9.1E-03	0.01	3.6E-03	100.0	Man-6-P biosynthesis
GNPTG	n.s.	n.s.	n.s.	n.s.	n.s.	n.s.	n.s.	Man-6-P biosynthesis
HEXA	n.s.	n.s.	0.25	2.8E-05	0.14	6.0E-06	7.1	terminal HexNAc degradation
HEXB	n.s.	n.s.	0.32	5.0E-05	0.28	1.1E-04	3.6	terminal HexNAc degradation
Gene	HEK293F	p-value	NK92	p-value	YTS	p-value	Magnitude of the maximum change	Enzyme Product
MAN2A2	n.s.	n.s.	< 0.01*	1.0E-06	0.24	1.0E-06	100.0	GlcNAc Man3 glycoform
MAN1C1	n.s.	n.s.	0.01	1.0E-06	0.02	1.0E-06	100.0	Man8 glycoform
MAN1A1	0.5	2.6E-02	0.33	5.7E-03	0.09	1.4E-03	11.1	Man5 glycoform
MAN2A1	0.66	5.1E-03	0.14	1.0E-06	0.13	1.0E-06	7.7	terminal α1-3 and α1-6Man hydrolysis in Man5 glycoform
MAN1B1	n.s.	n.s.	0.25	2.0E-06	0.16	1.0E-06	6.3	Man8 glycoform
MAN2B1	n.s.	n.s.	0.27	5.0E-06	0.22	4.4E-05	4.5	catabolism of N-linked oligosaccharides
MAN1A2	n.s.	n.s.	n.s.	n.s.	n.s.	n.s.	n.s.	Man5 glycoform
MANBA	n.s.	n.s.	0.1	5.8E-04	0.09	5.2E-04	11.1	βMan hydrolysis from non-reducing end of N-glycoproteins
MGAT4A	0.09	6.6E-04	< 0.01*	4.0E-04	< 0.01*	4.1E-04	100.0	Branch (β1-4GlcNAc to α1-3Man)
MGAT5	n.s.	n.s.	0.05	2.0E-04	< 0.01*	1.5E-05	100.0	Branch (β1-6GlcNAc to α1-6Man)
MGAT3	65.43	3.3E-05	n.s.	n.s.	0.31	8.1E-02	65.4	bisecting GlcNAc
MGAT4C	37.46	5.0E-06	0.26	3.2E-05	n.s.	n.s.	37.5	Branch (β1-4GlcNAc to a β1-2GlcNac-α1-3Man)
MGAT5B	34.38	1.2E-04	0.26	4.8E-02	23.89	6.9E-03	34.4	Branch (β1-6GlcNAc to β1-6Man and α1-3Man)
MGAT2	n.s.	n.s.	0.59	8.3E-03	0.27	9.4E-04	3.7	Second branch (GlcNAc Man5) in complex-type N-glycans

MGAT1	0.53	5.7E-04	0.68	2.9E-03	0.62	1.1E-03	1.9	First branch (GlcNAc Man5) in complex-type N-glycans
MGAT4B	1.56	4.4E-02	0.65	2.7E-04	0.6	4.0E-05	1.7	Branch (β1-4GlcNAc to a β1-2GlcNac-α1-3Man)
MOGS	n.s.	n.s.	0.44	7.0E-06	0.16	1.0E-06	6.3	distal α1-2Glu hydrolysis from Man9 glycoform
NAGPA	0.43	2.2E-02	0.32	4.4E-04	0.08	7.8E-05	12.5	Man-6-P biosynthesis
NEU4	0.28	4.1E-02	0.08	1.6E-02	0.1	2.4E-02	12.5	terminal α2-3, α2-6, and α2-8Neu5Ac hydrolysis
NEU1	4.41	6.2E-03	n.s.	n.s.	0.39	2.0E-04	4.4	terminal α2-3 and α2-6Neu5Ac hydrolysis
NEU2	n.s.	n.s.	0.26	2.8E-05	n.s.	n.s.	4.3	terminal α2-3, α2-6, and α2-8Neu5Ac hydrolysis

Gene	HEK293F	p-value	NK92	p-value	YTS	p-value	Magnitude of the maximum change	Enzyme Product
NEU3	1.85	3.6E-03	0.82	1.7E-02	0.51	2.0E-03	2.0	terminal α2-3, α2-6, and α2-8Neu5Ac hydrolysis
PRKCSH	n.s.	n.s.	0.35	4.9E-04	0.26	2.2E-04	3.8	α1-3Glu hydrolysis from Man9 glycoform
ST6GAL1	0.29	1.6E-04	0.12	4.0E-06	0.3	2.5E-05	8.3	α2,6 Neu5Ac
ST3GAL1	0.31	2.4E-04	0.22	1.0E-06	0.4	1.6E-04	4.5	α2,3 Neu5Ac
ST3GAL2	0.64	3.3E-03	0.4	1.7E-05	0.68	6.1E-03	2.5	α2,3 Neu5Ac
ST6GALNAC1	45.36	1.8E-04	0.36	5.1E-05	n.s.	n.s.	45.4	α2,6Neu5Ac to O-glycoproteins
ST8SIA2	368.06	9.2E-05	0.26	3.2E-05	n.s.	n.s.	368.1	polysialic acid
ST8SIA6	0.06	5.3E-03	0.01	3.6E-03	0.07	5.2E-03	100.0	polysialic acid
ST8SIA4	0.2	1.2E-02	1.9	7.7E-03	0.33	3.4E-02	5.0	polysialic acid
ST8SIA3	n.s.	n.s.	0.26	3.2E-05	n.s.	n.s.	4.3	polysialic acid
UGGT2	12.08	1.3E-03	n.s.	n.s.	0.01	9.0E-03	100.0	Protein folding quality control ER-associated reglucosylation
UGGT1	n.s.	n.s.	0.29	1.1E-04	0.16	7.4E-05	6.3	Protein folding quality control ER-associated reglucosylation

* values indicate very low abundance.

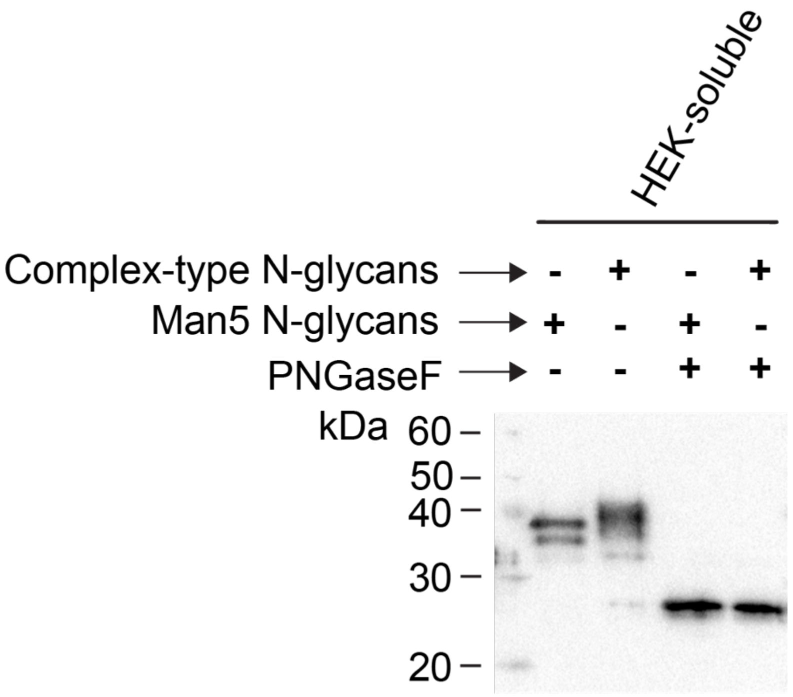

Figure S1. Expression of HEK-soluble CD16a using two different cell lines produces protein with predominantly oligomannose (Man5) N-glycans (HEK293S) or complex type N-glycans (HEK293F). Digestion with PNGase F indicates no differences in polypeptide mobility following N-glycan removal, though the polypeptide does migrate more slowly than expected based on mass (20 kDa).

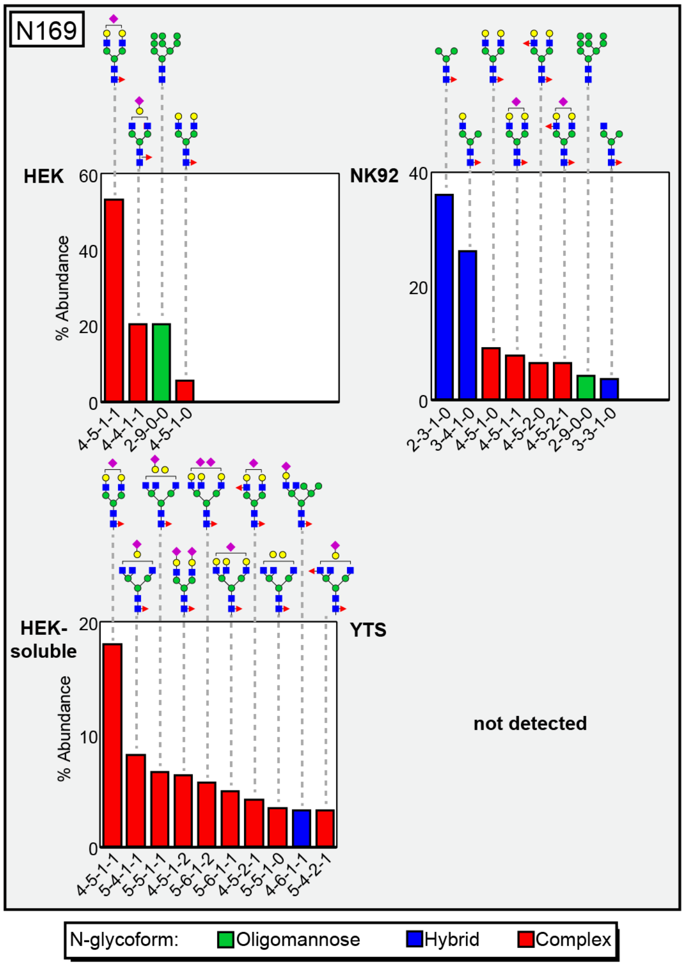

Figure S2. The top ten most abundant glycoforms at the CD16a N169 site. Cartoon diagrams represent one possible configuration; isobaric ions were not distinguished. The composition of each N-glycan is provided below the chart with numbers of N-acetylhexosamine, hexose, deoxyhexose, and N-acetylneuraminic acid residue, respectively.

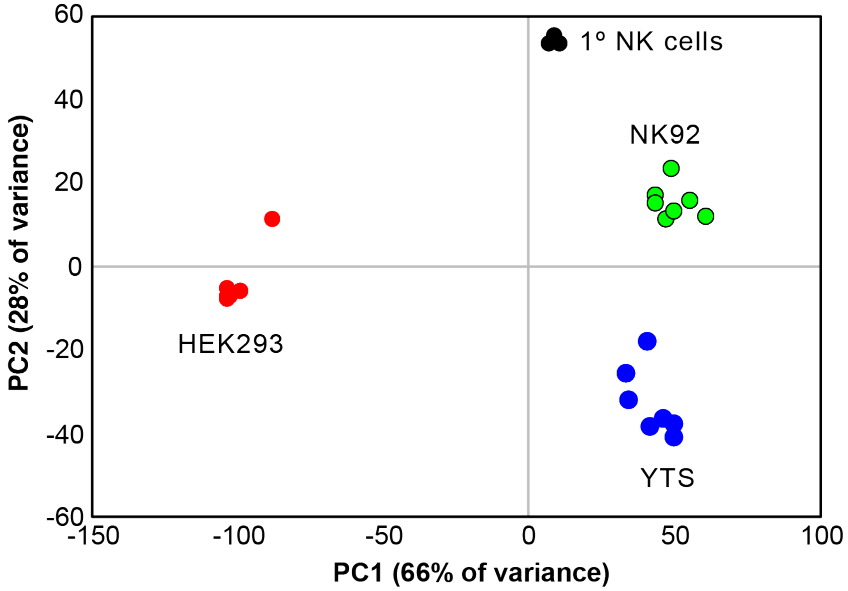

Figure S3. The four different cell types cluster based on the top two principle components of the RNA-Seq analysis.

Glycopeptide mass spectrometry parameter file

Method of Q Exactive

OVERALL METHOD SETTINGS

Global Settings	
Use lock masses	best
Lock mass injection	–
Chrom. peak width (FWHM)	10 s
Time	
Method duration	80.00 min
Customized Tolerances (+/-)	
Lock Masses	–
Inclusion	–
Exclusion	10.0 ppm
Neutral Loss	–
Mass Tags	–
Dynamic Exclusion	–

Experiment

FULL MS / DD-MS² (TOPN)	
General	
Runtime	0 to 80 min
Polarity	Positive
In-source CID	0.0 eV
Default charge state	2
Inclusion	–
Exclusion	on
Tags	–
Full MS	
Microscans	1
Resolution	70,000
AGC target	5e5
Maximum IT	80 ms
Number of scan ranges	1
Scan range	600 to 2500 m/z
Spectrum data type	Profile
dd-MS² / dd-SIM	
Microscans	1
Resolution	17,500
AGC target	2e5
Maximum IT	120 ms
Loop count	20
MSX count	1
TopN	20
Isolation window	2.0 m/z
Isolation offset	0.0 m/z
Scan range	200 to 2000 m/z
Fixed first mass	–
(N)CE / stepped (N)CE	nce: 17, 27, 37
Spectrum data type	Profile
dd Settings	
Minimum AGC target	2.00e3
Intensity threshold	1.7e4
Apex trigger	–
Charge exclusion	unassigned, 1, 6 - 8, >8
Peptide match	Off
Exclude isotopes	on
Dynamic exclusion	–
If idle ..	do not pick others

Setup

TUNEFILES
General
Switch Count 0
Base Tunefile C:\Xcalibur\methods\Nano_packedEmitter.mstune

CONTACT CLOSURE
General

Used	False
Start in Closed	True
Switch Count	0

SYRINGE
General

Used	False
Start in OFF	True
Stop at end of run	False
Switch Count	0
Pump setup	
Syringe type	Hamilton
Flow rate	3.000 µL/min
Inner diameter	2.303 mm
Volume	250 µL

DIVERT VALVE A
General

Used	False
Start in 1-2	True
Switch Count	0

DIVERT VALVE B
General

Used	False
Start in 1-2	True
Switch Count	0

LOCK MASSES
(no entries)

INCLUSION LIST
(no entries)

EXCLUSION LIST
41 entries

Mass [m/z]	Formula [M]	Species	CS [z]	Polarity	Start [min]	End [min]	Comment
1022.00000			1	Positive	0	80.00	
1122.00000			1	Positive	0	80.00	
1221.99000			1	Positive	0	80.00	
1321.99000			1	Positive	0	80.00	
1421.99000			1	Positive	0	80.00	
1521.99000			1	Positive	0	80.00	
1621.99000			1	Positive	0	80.00	
708.51000			1	Positive	0	80.00	
736.54000			1	Positive	0	80.00	
633.45000			1	Positive	0	80.00	
314.12000			1	Positive	0	80.00	
530.35000			1	Positive	0	80.00	
358.20000			1	Positive	0	80.00	
291.04000			1	Positive	0	80.00	
294.00000			1	Positive	0	80.00	

316.12000	1	Positive	0	80.00
338.34000	1	Positive	0	80.00
358.20000	1	Positive	0	80.00
376.26000	1	Positive	0	80.00
387.19000	1	Positive	0	80.00
390.14000	1	Positive	0	80.00
404.22000	1	Positive	0	80.00
421.32000	1	Positive	0	80.00
432.25000	1	Positive	0	80.00
460.28000	1	Positive	0	80.00
472.28000	1	Positive	0	80.00
508.27000	1	Positive	0	80.00
528.34000	1	Positive	0	80.00
551.63000	1	Positive	0	80.00
565.57000	1	Positive	0	80.00
610.62000	1	Positive	0	80.00
663.45000	1	Positive	0	80.00
684.20000	1	Positive	0	80.00
758.22000	1	Positive	0	80.00
761.22000	1	Positive	0	80.00
293.20000	1	Positive	0	80.00
550.63000	1	Positive	0	80.00
680.48000	1	Positive	0	80.00
269.06000	1	Positive	0	80.00
436.34000	1	Positive	0	80.00
313.14000	1	Positive	0	80.00

NEUTRAL LOSSES
(no entries)

MASS TAGS
(no entries)

Peptide mass spectrometry parameter file

Method of Q Exactive

OVERALL METHOD SETTINGS

Global Settings	
Use lock masses	best
Lock mass injection	–
Chrom. peak width (FWHM)	10 s
Time	
Method duration	80.00 min
Customized Tolerances (+/-)	
Lock Masses	–
Inclusion	–
Exclusion	10.0 ppm
Neutral Loss	–
Mass Tags	–
Dynamic Exclusion	–

Experiment

FULL MS / DD-MS² (TOPN)

General	
Runtime	0 to 80 min
Polarity	Positive
In-source CID	0.0 eV
Default charge state	2
Inclusion	–

Exclusion	on
Tags	—
Full MS	
Microscans	1
Resolution	70,000
AGC target	5e5
Maximum IT	80 ms
Number of scan ranges	1
Scan range	266 to 2500 m/z
Spectrum data type	Profile
dd-MS^2 / dd-SIM	
Microscans	1
Resolution	17,500
AGC target	2e5
Maximum IT	120 ms
Loop count	20
MSX count	1
TopN	20
Isolation window	2.0 m/z
Isolation offset	0.0 m/z
Scan range	200 to 2000 m/z
Fixed first mass	—
(N)CE / stepped (N)CE	nce: 27
Spectrum data type	Profile
dd Settings	
Minimum AGC target	2.00e3
Intensity threshold	1.7e4
Apex trigger	—
Charge exclusion	unassigned, 1, 6 - 8, >8
Peptide match	Off
Exclude isotopes	on
Dynamic exclusion	—
If idle ..	do not pick others

Setup

TUNEFILES
General
Switch Count 0
Base Tunefile C:\Xcalibur\methods\Nano_packedEmitter.mstune

CONTACT CLOSURE
General

Used	False
Start in Closed	True
Switch Count	0

SYRINGE
General

Used	False
Start in OFF	True
Stop at end of run	False
Switch Count	0
Pump setup	
Syringe type	Hamilton
Flow rate	3.000 μL/min
Inner diameter	2.303 mm
Volume	250 μL

DIVERT VALVE A
General

Used	False
Start in 1-2	True

Switch Count 0

DIVERT VALVE B

General

Used	False
Start in 1-2	True
Switch Count	0

LOCK MASSES

(no entries)

INCLUSION LIST

(no entries)

EXCLUSION LIST

41 entries

Mass [m/z]	Formula [M]	Species	CS [z]	Polarity	Start [min]	End [min]	Comment
1022.00000			1	Positive	0	80.00	
1122.00000			1	Positive	0	80.00	
1221.99000			1	Positive	0	80.00	
1321.99000			1	Positive	0	80.00	
1421.99000			1	Positive	0	80.00	
1521.99000			1	Positive	0	80.00	
1621.99000			1	Positive	0	80.00	
708.51000			1	Positive	0	80.00	
736.54000			1	Positive	0	80.00	
633.45000			1	Positive	0	80.00	
314.12000			1	Positive	0	80.00	
530.35000			1	Positive	0	80.00	
358.20000			1	Positive	0	80.00	
291.04000			1	Positive	0	80.00	
294.00000			1	Positive	0	80.00	
316.12000			1	Positive	0	80.00	
338.34000			1	Positive	0	80.00	
358.20000			1	Positive	0	80.00	
376.26000			1	Positive	0	80.00	
387.19000			1	Positive	0	80.00	
390.14000			1	Positive	0	80.00	
404.22000			1	Positive	0	80.00	
421.32000			1	Positive	0	80.00	
432.25000			1	Positive	0	80.00	
460.28000			1	Positive	0	80.00	
472.28000			1	Positive	0	80.00	
508.27000			1	Positive	0	80.00	
528.34000			1	Positive	0	80.00	
551.63000			1	Positive	0	80.00	
565.57000			1	Positive	0	80.00	
610.62000			1	Positive	0	80.00	
663.45000			1	Positive	0	80.00	
684.20000			1	Positive	0	80.00	
758.22000			1	Positive	0	80.00	
761.22000			1	Positive	0	80.00	
293.20000			1	Positive	0	80.00	
550.63000			1	Positive	0	80.00	
680.48000			1	Positive	0	80.00	
269.06000			1	Positive	0	80.00	
436.34000			1	Positive	0	80.00	
313.14000			1	Positive	0	80.00	

NEUTRAL LOSSES

(no entries)

MASS TAGS
(no entries)
